# Genetic Contributions to Prostate Cancer Disparities in Men of West African Descent

**DOI:** 10.3389/fonc.2021.770500

**Published:** 2021-11-08

**Authors:** Jabril R. Johnson, Leanne Woods-Burnham, Stanley E. Hooker, Ken Batai, Rick A. Kittles

**Affiliations:** ^1^ Division of Health Equities, Department of Population Sciences, City of Hope Comprehensive Cancer Center, Duarte, CA, United States; ^2^ Department of Urology, University of Arizona, Tucson, AZ, United States

**Keywords:** genetics, prostate cancer genetics, prostate cancer disparities, GWAS, Vitamin D, prostate cancer screening, socioeconomic status, precision medicine

## Abstract

Prostate cancer (PCa) is the second most frequently diagnosed malignancy and the second leading cause of death in men worldwide, after adjusting for age. According to the International Agency for Research on Cancer, continents such as North America and Europe report higher incidence of PCa; however, mortality rates are highest among men of African ancestry in the western, southern, and central regions of Africa and the Caribbean. The American Cancer Society reports, African Americans (AAs), in the United States, have a 1.7 increased incidence and 2.4 times higher mortality rate, compared to European American’s (EAs). Hence, early population history in west Africa and the subsequent African Diaspora may play an important role in understanding the global disproportionate burden of PCa shared among Africans and other men of African descent. Nonetheless, disparities involved in diagnosis, treatment, and survival of PCa patients has also been correlated to socioeconomic status, education and access to healthcare. Although recent studies suggest equal PCa treatments yield equal outcomes among patients, data illuminates an unsettling reality of disparities in treatment and care in both, developed and developing countries, especially for men of African descent. Yet, even after adjusting for the effects of the aforementioned factors; racial disparities in mortality rates remain significant. This suggests that molecular and genomic factors may account for much of PCa disparities.

## Introduction

It is estimated that 1 in 9 men in the United States (U.S.) will be diagnosed with prostate cancer (PCa) in their lifetime (1). However, the estimation rate for African American (AA) men is 1 in 7, and this rate may be higher in AAs as less cases have been detected in recent years due to fluctuations in screening guidelines (2). In a recent cross-sectional study, it was reported that 1 in 3 AA men living in Southern California and New York City had elevated circulating prostate-specific antigen (PSA) (3). Not surprisingly, this elevated rate of PCa diagnosis in AA men also correlates with the highest disease-specific mortality rates for this population (1). The factors contributing to the glaring disparities in incidence and outcome are multifaceted and include sociocultural (e.g., behavioral), socio-political (e.g., racism and discrimination), and biological (e.g., genetic risk, biomarkers, genetic ancestry) (4). Yet, even after adjusting for the effects of the non-biological determinant factors; racial disparities in mortality rates remain significant. This suggests biological determinants (e.g., molecular and genomic factors) may contribute greatly to PCa disparities (5–7). Further exacerbating the confounding variables, blanket screening recommendations for PCa have fluctuated over the past decade. Controversy remains regarding the pros and cons of modified screening guidelines specific to race or family history. Numerous studies highlight the advantage of earlier and annual screening for AA men, however these findings have yet to translate into clinical application (8, 9). In fact, as described in the aforementioned study, more than half of AA male study participants reported that their physicians had never discussed the pros and cons of PCa screening with them. Of concern, one-third of the same men had elevated PSA levels at the time of the study (3).

In addition to the lower rates of screening and higher rates of diagnosis experienced by AA men, the biological characteristics of prostate tumors are worse at clinical presentation for this population (10). Delayed diagnosis limits treatment options and reduces the efficacy of those treatments which contributes to worse overall survival. While cutting-edge treatment options are on the horizon for currently incurable advanced PCa, AAs are less likely to participate in clinical trials for a multitude of reasons including reduced study-initiated recruitment efforts and medical mistrust within this population (11). This review will address the complex interplay of factors which drive the continued disparities experienced by AA men with PCa as well as provide future direction on how to potentially reverse these troubling trends.

## Prostate Cancer Incidence and Mortality Rates

**Figure 1:**
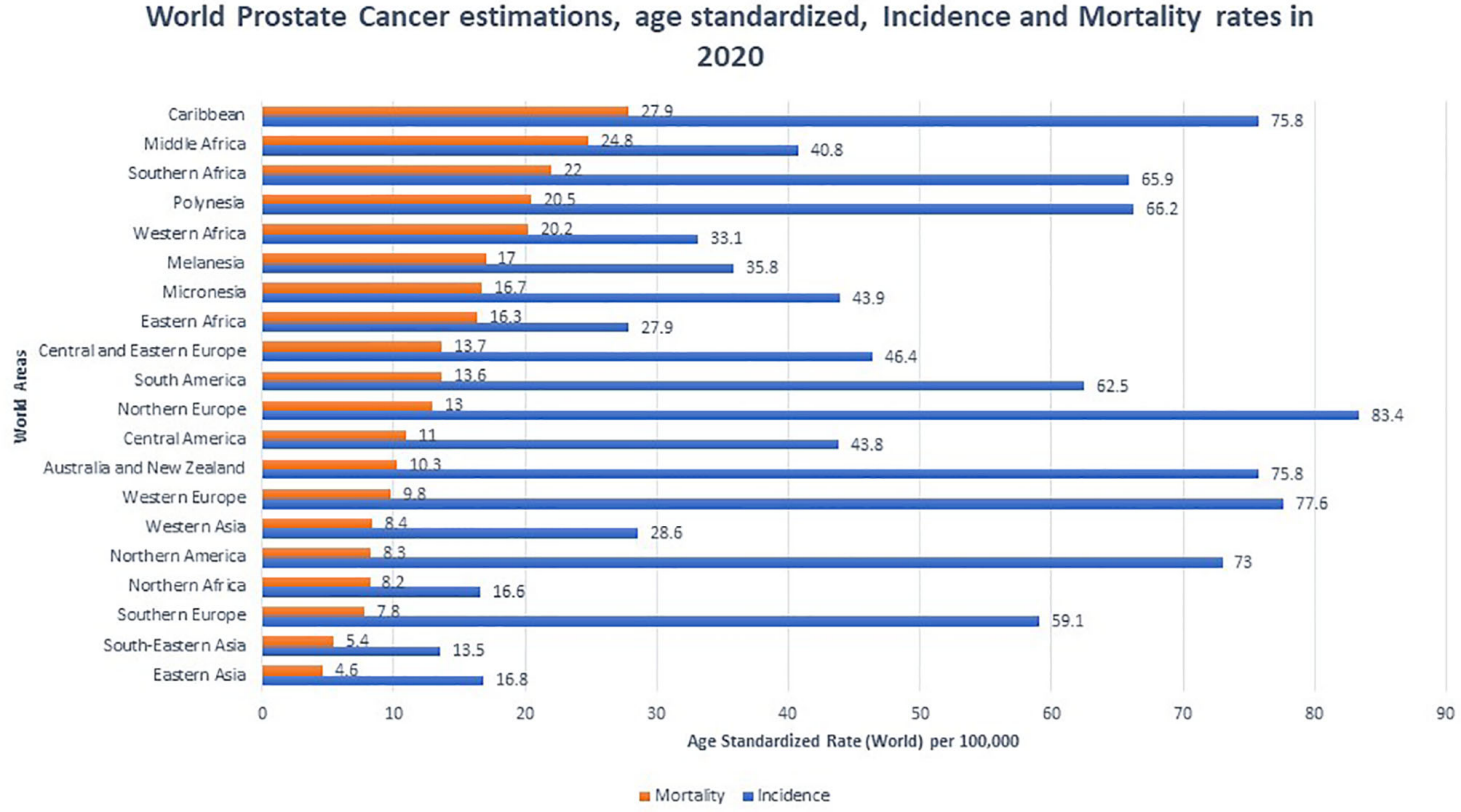
Global prostate cancer incidence and mortality by World Areas. Graph indicates age standardized incidence (Blue) and mortality rates (Orange), per 100,000. Mortality rates were sorted from highest to lowest (12).

PCa is the most common non-cutaneous malignancy in men worldwide. Rates of PCa incidence and mortality vary between population, and developed nations tend to have higher incidence while developing nations tend to have higher mortality rates (12) (**Figure 1**). Lower mortality rates in developed nations are attributed to increased PSA testing, greater health literacy, and better access to health care. In developing nations, advanced PCa disease and worse prognosis are due to lack of access to screening which is proportionate to increased detection of late stage prostate carcinoma (13). In 2020, Africa (South, East, Middle and West) and the Caribbean suffered the highest PCa mortality in the world with rates ranging from 16.3-27.9 deaths per 100,000 (12). Interestingly, although North America has a low mortality rate, when stratified by race and adjusted for age, AAs had a similarly high mortality rate of 37.4 per 100,000 in the period of 2014-2018. This number is significantly higher than non-Hispanic whites (19.3 per 100,000), Hispanics (15.6 per 100,000), American Indian (18.5 per 100,000), and Asian/Pacific Islanders (8.8 per 100,000) (14).

### Prostate Cancer Screening Recommendations: One Size Does Not Fit All

Prostate cancer is often indolent or slow growing and preventable if diagnosed and treated early. However, if left untreated, can lead to severe disease progression (i.e. bony osteopathic metastasis) and often time poor prognosis. Digital Rectal Exams (DRE), which may identify hard nodules on and around the prostate gland and elevated Prostate Specific Antigen (PSA) values, are often used to screen for potential prostate cancer. Confirmation of the diagnosis of prostate cancer is made by histologic and biopsy assessment of biopsy tissue using a Gleason grading and TMN staging system (15).

Since the early 1990s, the measurement of total circulating prostate-specific antigen (PSA) along with a digital rectum exam (DRE) have been mainstay tools for PCa screening. Common screening practices use a higher-than-normal PSA cutoff of >4.0 ng/mL to recommend a follow-up biopsy. However, in 2004 the Prostate Cancer Prevention Trial (PCPT) challenged this standard by reporting that PCa diagnosed with a Gleason score 7 or higher was detected in 15% of men with PSA <4.0 ng/mL. In addition, PCa was detected in men whose PSA ranged between 3.0–4.0 ng/mL (16). Therefore, data suggests PSA levels should be measured and interpreted as continuous risk; as PSA levels increase, the likelihood of PCa diagnosis increases. However, although there is a correlation between increased PSA levels and presence of PCa, it must be noted that PSA levels >4.0 ng/mL are not diagnostic for PCa. In fact, there are several factors that impact PSA level fluctuation in men including an enlarged prostate (benign prostatic hyperplasia (BPH)), age, prostatitis, ejaculation, certain urologic procedures, and certain medications (17).

As a result of multiple factors stimulating total PSA fluctuation and confounding interpretations of what PSA levels may imply, screening for PCa has been a challenge. In 2012, the United States Preventive Services Task Force (USPSTF) issued concerns about the risk of possible untoward side effects due to unnecessary biopsies, overdiagnosis, and overtreatment of screen-detected, indolent tumors which led to a Grade D recommendation against blanket PSA-based screening for PCa (18). The stance of the USPSTF was later amended in 2018 to a Grade C recommendation for men between 55-69 to be offered the opportunity to discuss the potential benefits and harms of screening with their clinicians (19). In response, the American Urological Association (AUA) commended the USPSTF recommendations and acknowledged that AA men are at increased risk of developing the disease. The American Cancer Society took a more proactive stance regarding earlier screening in AA men and subsequently recommended screening at age 45 for AA men, and age 40 for AA men with extensive family history, with repeat annual PSA testing for any man whose levels exceed 2.5 ng/mL (20). The Prostate Cancer Foundation also erred on the side of caution and recommended screening for all men at age 45, and age 40 for AA men as well as any man with family history (21). Given the amended guidelines debating the effectiveness of measuring total PSA, as a biomarker for PCa, additional metrics have also been implemented to enhance the specificity and sensitivity of PSA. PSA screening enhancements include PSA velocity, PSA density, free *vs*. bound PSA, proenzyme PSA (ProPSA), prostate health index (PHI), and the 4K (Four-Kallikrein) score test (15).

**Figure 2:**
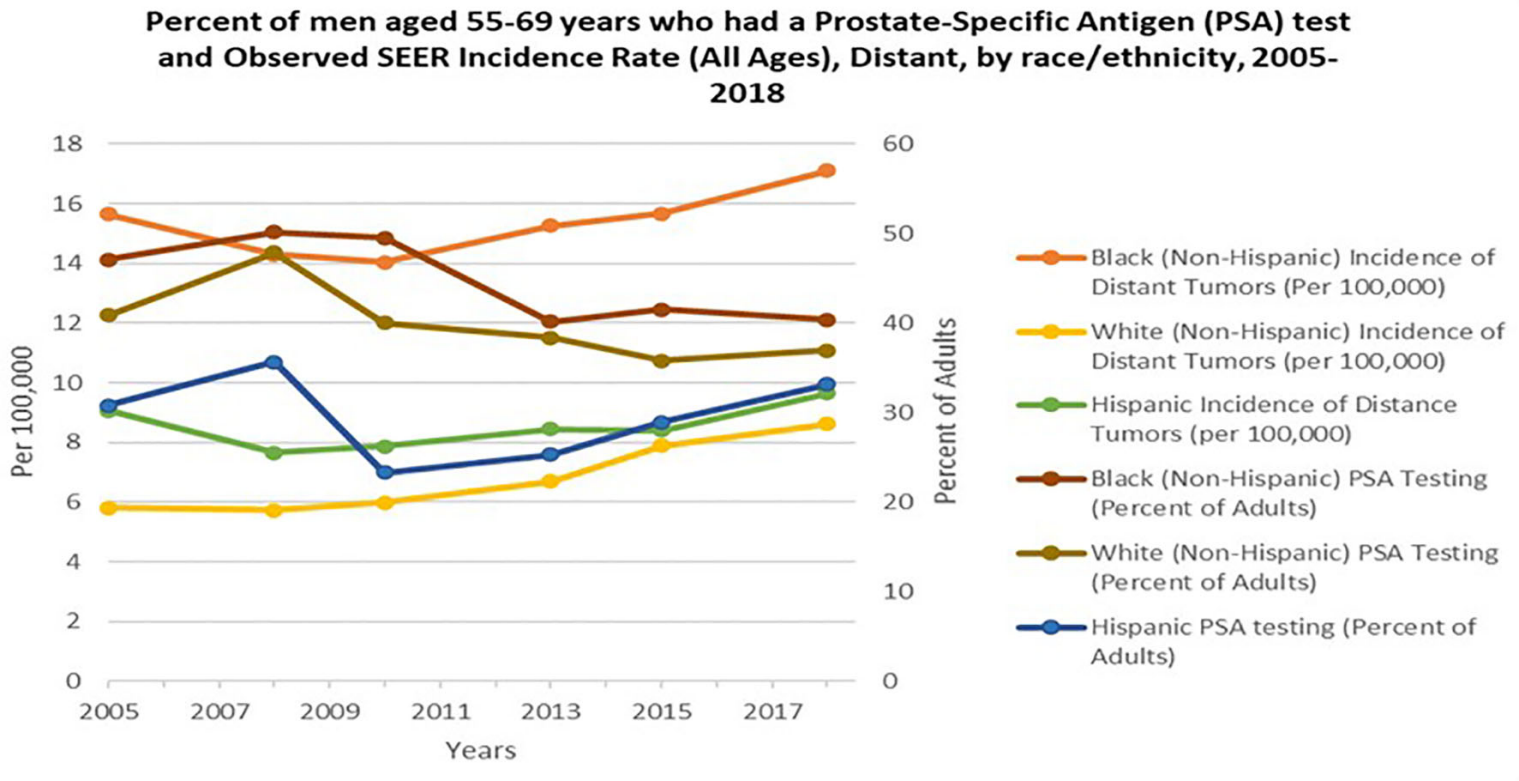
Percent of men aged 55-69 years who had a Prostate- Specific Antigen (PSA) test and Observed SEER Incidence Rate (All Ages), Distant, by race/ethnicity, 2005-2018. Orange, Yellow and Green lines represent Black, NHW (non-Hispanic white) and Hispanic incident of distant tumors per 100,000 persons in the U.S, respectively. Burgundy, Brown, and Blue lines represent Black, NHW (non-Hispanic white) and Hispanic PSA testing percent of adults in the U.S. Data adapted from Centers for Disease Control and Prevention, National Center for Health Statistics, National Health Interview Survey, 2005-2018 and SEER 21 areas estimates for a specific stage at diagnosis (Localized, Regional, Distant or Unknown/Unstaged) (29, 30).

Widespread PSA screening has had a profound effect on identifying PCa at earlier stages before incurable metastasis occurs (22). This is supported by the decrease in mortality rate by greater than 53% during the PSA screening era (1991-2008), once adjusted for age (23). Cancer Intervention and Surveillance Modeling Network (CISNET) estimated that 45-70% of this decrease was attributed to PSA screening (22). It is plausible that, although PCa disparity may be impacted by socioeconomic circumstances and lifestyle decisions, intensive PSA screening and comprehensive PCa edification may mitigate PCa disparities in the U.S. and throughout the African diaspora (24, 25). In African populations, where minimal PSA screening is available, Burkina Faso, Ghana and Port Harcourt Nigeria, men with high serum PSA levels and poorly differentiated tumors are major characteristics reported at the time of diagnosis (13). Likewise, Gueye and colleagues found increased median and mean PSA levels and worse tumor stage among Senegalese men compared to EA and AA men (26). Numerous PCa studies focused on the African diaspora and in the U.S. suggest the need for updated PCa screening protocols. Yet, the benefits of PSA screening and the direct implications it may have in mitigating the PCa disparity gap in the U.S. remain controversial. In 2018, the USPSTF PCa screening guidelines stated there was inadequate evidence to assess whether the benefits for high-risk AAs were different than the benefits for average-risk populations. The guidelines also emphasized inadequate evidence to assess whether screening benefits high-risk groups younger than 55 years of age. Noticeably, the USPSTF stance is contrary to compelling evidence of the increased burden of PCa morbidity and mortality in these populations (27). Several studies have shown higher serum PSA levels and higher PSA density in AAs than EA’s. Preston and colleagues reported that young AAs, ages 20-45 years, presented with higher baseline serum PSA levels than young EA men (28). Taken together, the USPSTF recommendation engenders major complications and a hazard to men of African descent. This is largely due to the recommendation being predicated on results from the Prostate, Lung, Colorectal and Ovarian Cancer Screening Trial (PLCO) and the European Randomized Study of Screening for Prostate Cancer (ERSPC). The PLCO reported only 4% of enrolled men were non-Hispanic Black, while the ERSPC did not report demographic statistics. Thus, the low sample sizes of men of African descent in the PLCO and ERSPC study lends deserving cynicism to the USPSTF recommendations for these high-risk populations (25). Overall, although the USPSTF recommendation of decreasing PSA testing in 2012 may have been of cost benefit in avoiding unnecessary biopsies, not making specialized considerations for AA men that are twice as likely to be diagnosed and three times as likely to die from PCa, is harmful and irresponsible. Hence, after the 2012 recommendation for PSA screening to decrease, incidence of distant tumors or aggressive presentation for AA men significantly increased (**Figure 2**) (29, 30).

## Socioeconomic Factors: Barriers That Impact Equity of Pca Treatment For African Americans

Elucidating the complexities that underpin the PCa continuum (i.e. risk, progression and aggressive presentation), has emphasized the importance of non-biological determinants (i.e. socioeconomic status) role in PCa disparity. Compelling evidence has also implicated the role of socioeconomic status, which is a composition of: social environment constructs (i.e. resident population, crime, community support, social capital, racial segregation, presence of trash and graffiti) and man-made physical environmental attributes (i.e. structural conditions that affect access and availability to health promoting resources and influence individual health behaviors), to be associated with PCa risk and advanced presentation in AA men (31, 32). Though, AA men tend to have lower SES (socioeconomic status) (i.e. education, income, (29) wealth and neighborhood socioeconomic status) than EA men (29, 33, 34) differences in SES may present as barriers that impact PCa treatment and overall outcomes (35). For example, Watson and colleagues designed a cross-sectional analysis of AA and Non-Hispanic White (NHW) men (n=2,386) sampled from the Pennsylvania Cancer Registry combined with neighborhood census data (P^2^ Access study), to investigate the role of SES on PCa treatment. The authors concluded that men living in neighborhoods with higher SES (i.e. wealth, education, access to healthcare) were more than likely to receive definitive treatment (OR 1.57, 95% CI 1.01, 2.42), compared to those living in less advantaged neighborhoods (36). To not oversimplify the results of the study, it is plausible to infer higher education led to more informed decisions and increased wealth led to increased access to options and treatment. DeRouen and colleagues reported similar findings in a population-based case-control study from the San Francisco Bay Area Prostate Cancer study. Authors performed a multi-level data analysis (including education and known PCa risk factors) to investigate the unique interplay between the socio and man-made physical environment on (37) localized and advanced PCa presentation, between AA and NHW. Interestingly, education was not associated with localized PCa, but greater education was associated with lower risk of advanced disease. Higher education was also protective for advanced PCa for men living in low SES neighborhoods but not for men living in high SES neighborhoods. Lastly, lower neighborhood SES association with greater localized disease was explained by known PCa risk factors as well as neighborhood environmental factors (i.e. population density, crowding, and residential mobility) (37). In contrast, previous studies have reported higher neighborhood SES being associated with higher PCa incidence (38). Suggesting greater access to healthcare and more regular PSA screenings, contributed to higher rates of PCa incidence in those living in populated urban areas.

Nonetheless, historically, the PCa disparity gap has been attributed to the fact that AA men are less likely to be screened for PCa (24). However, many reasons have been proposed to explain why AA men receive less screening, including but not limited to: poor communication between physicians and minority patients, lack of access to health care, stigmatization/fear and deficiency of knowledge about screening (25, 39, 40). Although there is increasing awareness, many AA men remain uninformed of the current early detection methods available for PCa (e.g., PSA testing), which is accounted for in the aforementioned studies regarding lower education being correlated to increased advanced PCa. Other barriers such as cost and transportation may additionally hinder some AA men from being screened (41). Minimizing differences in quality healthcare availability may be a potentially important pathway to minimizing disparities in PCa outcomes. Moreover, the relationships between socioeconomic factors and PCa are complex; but AA men have high PCa mortality in all socioeconomic groups or accounting for socioeconomic factors suggesting that genetic and biologic factors contribute to the disparities in PCa mortality (32, 42, 43).

## Genome-Wide Association Studies and Admixture Mapping

Genome wide association studies (GWAS) have revolutionized the field of cancer genetics over the past decade. Specifically, the field of PCa genetics has benefited immensely from GWAS methodologies. This improved understanding is largely due to the elucidation of key genes and gene variants involved in a myriad of molecular and biological mechanisms that propel PCa etiology. Although men of African descent have increased PCa incidence and mortality rates, most large scale GWAS have been conducted in European populations (44). This trend engendered the need to conduct original GWAS for African men and other men of African descent as well as replicate and validate PCa risk loci and variants previously identified in GWAS of men of African descent. According to the National Human Genome Research Institute-European Bioinformatic Institute GWAS catalogue database (45), only 10 of 65 (15%) of GWAS prostate carcinoma studies included African men and/or men of African descent. Fortunately, the few studies that included African men and men of African descent in the initial or replicate samples has allowed the detection of PCa susceptibility variants associated with African ancestry (46–48) West African ancestry (WAA).

In addition to GWAS, admixture mapping has also been used to identify genomic loci associated with PCa. One of the benefits of genetic studies with participants from admixed populations is the ability to leverage the heterogeneity of their ancestral chromosomal segments in the study of biomarkers and disease (49, 50). Admixture mapping is a statistical analysis that leverages the local genomic ancestry in admixed populations to identify genomic regions associated with disease - granted the disease prevalence varies in at least two of the ancestral groups that contribute to the genetic makeup of the admixed group under study. It has been used to identify genes and loci involved in several phenotypes and diseases, including white blood cell count and breast cancer (51, 52).

As previously stated, PCa has increased risk for incidence, aggressiveness, and mortality in African and African descent populations when compared to European descent populations, thus making PCa an optimal disease model for Admixture mapping analysis. There is evidence suggesting WAA is informative in predicting PCa diagnosis and aggressiveness. However, the complex relationships between genetic ancestry and social factors make the use of global genetic ancestry estimation, using ancestry informative markers located across the genome, difficult when we assess the contributions of ancestry-related genetic factors to PCa disparities (9, 53–55). The admixture mapping approach has been more successful and studies using admixture mapping have identified chromosomal segments of WAA in the 8q24 associated with PCa risk (56, 57). Enrichment of WAA in this region was significantly associated with elevated PCa risk, and each African-derived chromosomal segment in the region increased about 1.5-fold risk. Another admixture mapping study in AA men identified an additional locus at 5q35 region showing a significant association between WAA and PCa risk (5). In these studies, the associations were stronger in the younger age group suggesting ancestry-related factors contributing to early age onset of PCa in men of WAA. In the order AA men, enrichment of WAA on 3q13 region was associated with PCa risk (5). While admixture mapping is a statically powerful approach to identify candidate regions, it does not allow for identifications of specific variants contributing to disease risk.

Fine mappings through GWAS, on the other hand, allow us to identify PCa risk loci, and GWAS mainly conducted in European descent identified many PCa risk loci (48, 58–60). However, replications of GWAS findings in AA men has been challenging due to difference in linkage and risk allele frequency. Many GWAS identified PCa risk variants have greater allele frequency differences than expected from genome-wide average across Human populations. Thus, many studies are successful in replicating only a subset of GWAS identified variants (61, 62). We have analyzed the genome of 755 unrelated self-reported AA men (454 cases and 301 controls) and validated five single nucleotide polymorphisms (SNPs) (rs10896449 on *NUDT11 (Nudix Hydrolase)* 11q13.2 (p=.0009); rs2735839 on *KLK2*/*3* 19q33.33 region (p=.04); rs443076 on *HNF1B*/*TCF2 (Hepatocyte Nuclear Factor-1 Beta)* 17q12 (p=.008); rs5945572 on Xp11.22 (p=.05)) and a rare variant specific to WAA (bd11934905 in region 2 of 8q24 (p=1x10^-4)^) that were reported in previous GWAS. Although we were able to replicate a few of the previous GWAS variants, most were not able to be confirmed (63). The genetic variants that better capture PCa risk are located close by (64). Thus, supporting the premise, more GWAS and additional fine mapping in AAs are necessary to identify and confirm novel susceptibility loci.

Despite these challenges in replication studies, multiple studies have replicated the associations between the 8q24 variants and PCa risk in West African and West African descent populations. West African genetic ancestry has been found to be overrepresented in the 8q24 region in AA men with PCa (46). In the 8q24 regions, there are multiple variants and regions that are independently contributing to elevated PCa risk (65–67). Strong associations between the 8q24 and PCa have been shown not only in African Americans, but also in African descent men in Caribbean nations and West Africans (68–70). Risk allele frequencies in this region are higher in African descent populations compared to non-African populations. With WAA estimated for the 8q24 region, both the likelihood of having multiple 8q24 variant risk alleles and the odds of PCa diagnosis increase (54). In a GWAS meta-analysis, Conti and colleagues identified a low frequency variant in the 8q24, rs72725854, associated with more than 2-fold increased risk of PCa (71). The risk variant, rs72725854 T allele, has been found only in African descent populations with allele frequency ranging between 6% and 11%. Further evidence of African descent populations and genetic susceptibility to PCa development is supported by Du Z and colleagues who conducted an association study of >100 previously reported PCa risk alleles among 1,061 Ugandan men (571 cases and 485 controls) and tested associations of 17,125,421 genotyped and imputed markers genome wide for PCa risk. Data showed approximately 100 known PCa risk variants were associated with 10 percent of Ugandan men having >4-fold increased PCa risk. In addition, the 8q24 risk region was found to be a major contributor to PCa risk in Ugandan men (72). The African specific variant, rs72725854 T allele, was associated with more than 3-fold elevated odds. This variant also shows stronger associations in men of WAA with family history and diagnosed before the age of 60 years or younger (73). No gene has been identified with PCa in the 8q24 region, as this locus is a gene desert. However, subsequent fine mapping of this region has identified several long non-coding RNAs. The African specific variant rs72725854 is located at the enhancer region, and the risk allele is associated with higher expression of non-coding RNAs and MYC gene (74). Han and colleagues conducted fine mapping of the 8q24 risk region to identify novel associations with common and rare variation in 9,531 (4853 cases and 4678 controls) men of African descent. They identified three independent variants (*r^2^* <0.0018), all located near PCa long noncoding RNAs (lncRNAs), including *PRNCR1 (Prostate Cancer Associated non-coding RNA 1)*, *PCAT1 (Prostate Cancer Associated Transcript 1)* and *PCAT2 (Prostate Cancer Associated transcript 2)* (75). These are WAA-specific risk variants that exists only in WAA populations. Although these genes are non-coding, numerous studies have implicated dysfunction of lncRNAs in PCa etiology and progression (76–78).

Despite the challenges in replication of GWAS findings, studies have shown that GWAS identified PCa risk variants, particularly variants replicated in AA men and high odds ratios, are higher risk allele frequency in WAA populations (54, 79, 80). As observed for 8q24 variants, both likelihood of having multiple risk alleles and odds of PCa diagnosis and aggressive PCa increase with estimated WAA (54). Unlike many other PCa genetic susceptibility loci that lack replicative analysis, genetic variation in 8q24 has been consistently associated with PCa risk across populations and is highly prevalent in men of African descent. Taken together, genetic contribution of PCa risk, such as the 8q24 region, may play a critical role in PCa etiology in men of African descent. A complete list of GWAS and genetic PCa susceptibility loci, in African and men of African descent, can be found in (**Table 1**). Some of the novel risk variants other than 8q24 regions identified in a recent large scale GWAS meta-analysis are African ancestry specific risk variants. However, only a few studies conducted genome scan to identify PCa risk loci specifically in African populations (72, 81–83). Africa has great genetic diversity (84). Including more African men in GWAS and fine-mapping studies may identify additional WAA-specific risk variants.

**Table 1:** PCa associated genes reported from GWAS in African and men of African descent throughout the African Diaspora.

First Authors	Study Accession	Gene Locus Location	*Mapped Gene(s)*	Variant and Risk Allele	Discovery Sample Number and Ancestry	Replication Sample Number and Ancestry	Context
Haiman CA et al. (47)	GCST001078	17:49359387	*ZNF652*	rs7210100-A/2C	6,715 African American or Afro-Caribbean	3,408 African American or Afro-Caribbean; 1,705 Sub-Saharan African	Intron
Cook MB et al. (81)	GCST002264	1:18900554	*PAX7*	rs114799364-T	932 Sub-Saharan African	10,068 African American or Afro-Caribbean	5’ UTR
		1:57769516	*DAB1*	rs12057381-A			Intron
		2:14603290	*NCRNA00276 and FAM84A*	rs2056150-A			5’ UTR
		2:6085200	*LOC150622 (AC073479.1)*	rs13432692-T			Intron
		2:121081260	*AC012363.13 and INHBB*	rs12477565-T			Intron and 5’ UTR
		3:6935066	*GRM7-AS2 (AC066606.1)*	rs114246623-A			Intron
		5:140166953	*PCDHA1*	rs34575154-G			Exon-Missense
		5:140177075	*PCDHA1*	rs116776862-A			Intron
		5:140213805	*PCDHA1, PCDHA2, PCDHA3, PCDHA4, PCDHA5, PCDHA6*	rs4151685-C			Intron
		5:140711097	*PCDHGA1*	rs17097185-G			Exon-Missense
		5:140711954	*PCDHGA1*	rs116679801-G			Exon-Missense
		5:140718552	*PCDHGA1 and PCDHGA2*	rs6878145-G			Intron and Exon-Missense
		5:140718750	*PCDHGA1 and PCDHGA2*	rs61749035-T			Intron and Exon-Missense
		6:32655508	*AL662789.1 and HLA-DQB1*	rs115850745-G			3’ UTR
		6:40084864	*RP11-552E20.1 and MOCS1*	rs115899206-G			3’ UTR
		6:47663326	*GPR111*	rs1329536-T			3’ UTR
		7:67865802	*RP5-945F2.3 and STAG3L4*	rs73146440-G			5’ UTR
		8:3247408	*CSMD1*	rs147739031-G			Intron
		9:15111573	*U6.1033 and TTC39B*	rs10961884-C			5’ UTR and 3’UTR
		10:8462439	*GATA3 and RP11-543F8.2*	rs2993385-C			5’ UTR and Intron
		10:8474595	*GATA3 and RP11-543F8.2*	rs7918885-G			5’ UTR and Intron
		10:8479257	*GATA3 and RP11-543F8.2*	rs12251624-C			5’ UTR and Intron
		10:8479868	*GATA3 and RP11-543F8.2*	rs7090925-G			5’ UTR and Intron
		10:8480044	*GATA3 and RP11-543F8.2*	rs10905371-G			5’ UTR and Intron
		10:8481486	*GATA3 and RP11-543F8.2*	rs10905374-A			5’ UTR and Intron
		10:8484113	*GATA3 and RP11-543F8.2*	rs7096374-T			5’ UTR and Intron
		10:8486161	*GATA3 and RP11-543F8.2*	rs7896254-A			5’ UTR and Intron
		13:30562130	*LOC440131 (RP11-90M5.1)*	rs75404762-C			5’UTR
		20:42364734	*GTSF1L and MYBL2*	rs285198-A			3’UTR
		20:42371095	*GTSF1L*	rs370971-A			3’UTR
Shan J et al. (82)	GCST002024	9	*SMARCA2*	rs7045455-C	221 Greater Middle Eastern (Middle Eastern, North African or Persian)	438 Greater Middle Eastern (Middle Eastern, North African or Persian)	Intron
		9	*SMARCA2*	rs12686439-A			Intron
		9	*SMARCA2*	rs10810919-C			Intron
		9	*SMARCA2*	rs10963533-C			Intron
		9	*SMARCA2*	rs10963540-A			Intron
		17	*STAT5A*	rs12601982-G			Intron
		17	*STAT3*	rs8078731-T			Intron
		22	*LOC646851*	rs5750627-T			Intron
		22	*LOC646852*	rs6001173-T			Intron
		22	*SUN2 and GTPBP1*	rs138702-A			Intron
		22	*SUN2*	rs138712-G			Intron
Al Olama AA et al. (48)	GCST002606	1:205788696	*AC119673.1 and SLC41A1*	rs1775148-C	67,543 European; 2,080 Hispanic or Latin American; 6,954 East Asian; 10,463 Sub-Saharan African, African American or Afro-Caribbean	NR	NR
		6:75786165	*MYO6*	rs9443189-G			Intron
		14:60655808	*SIX1*	rs7153648-C			NR
		16:71657426	*PHLPP2 and AC009097.1*	rs12051443-A			Intron
		20:50911385	*ADNP*	rs12480328-T			Intron
		21:41529494	*TMPRSS2*	rs1041449-G			NR
		22:19770369	*TBX1*	rs2238776-G			Intron
Hoffmann TJ et al. (83)	GCST002944	19:50851341	*KLK3 and AC011523.1*	rs2659124-T	3,226 East Asian; 2,251 African American or Afro-Caribbean; 3,629 Hispanic or Latin American; 37,272 European	4,679 African American or Afro-Caribbean; 7,539 European	NR
		6:160160512	*AL645733.1 and SLC22A1*	rs4646284-TG			3’ UTR
Conti DV et al. (71)	GCST004982	13:109708437	*AL163541.1*	rs75823044-T	8,298 African unspecified, African American or Afro-Caribbean; 932 Sub-Saharan African; 11,782 African American or Afro-Caribbean	NR	NR
		22:27978955	*TTC28-AS1 and TTC28*	rs78554043-C			Intron and 3’ UTR
Du Z et al. (72)	GCST005786	1:224217786	*AC092809.4 and AC092809.2*	rs61005944-CT	1,040 Sub-Saharan African	NR	Indel/Upstream
		1:79987113	*AC099671.1*	rs1340678-T			NR
		1:89431556	*GBP1P1 and LRRC8B*	rs12095604-C			NR
		2:127104557	*BIN1*	rs6431219-C			Intron
		2:41094221	*LINC01794 and HNRNPA1P57*	rs148184576-A			NR
		2:97135631	*ANKRD36*	rs58488929-C			NR
		3:184083406	*HTR3C2P*	rs5855014-GC			Intron
		5:116942357	*AC010267.1 and AC093534.1*	rs76861935-C			Intron
		5:162565392	*AC113414.1*	rs17060512-T			Intron
		6:4905041	*CDYL*	rs79774606-G			Intron
		7:29103463	*CPVL*	rs6979813-C			Intron
		8:127062570	*PCAT1*	rs72725854-T			NR
		8:127164718	*PCAT1 and CASC19*	rs76784613-G			Non Coding
		9:11130398	*AL451129.1 and AKAP8P1*	rs2151715-G			NR
		9:12079333	*AL353595.1 and AL589678.1*	rs4741206-G			NR
		9:12184024	*AL589678.1 and JKAMPP1*	rs73408421-A			NR
		10:53578095	*RNA5SP318 and AL365496.1*	rs11003686-A			NR
		13:112620498	*AL139384.2 and ATP11AUN*	rs7325069-G			NR
		13:36861531	*SMAD9*	rs140971918-G			Intron
		14:97356528	*LINC02325 and AL158800.1*	rs234439-G			NR
		16:7552979	*RBFOX1*	rs112896149-G			Intron
		18:49679976	*SMUG1P1 and AC090227.2*	rs8093567-T			Intron
		19:44371829	*AC245748.1*	rs2571082-G			NR
		19:56687205	*AC006115.2 and ZIM2-AS1*	rs7258285-G			Intron

Associations reported are statistically significant at p<10^-8^ and validated in at least one independent sample (45).

## Hereditary Prostate Cancer Genes

Among all common cancer types, PCa has the highest heritability. Hereditary PCa has been defined as 1) a cluster of ≥3 first-degree relatives with PCa, 2) PCa in each of 3 generations, or 3) 2 relatives with PCa diagnosed before age 55 (85). Hereditary PCa accounts for approximately 10% of PCas and is generally associated with early onset disease (86). Linkage studies have been used to identify genes associated with hereditary PCa in families. Specifically, linkage analyses scan the genome using genetic variants or markers to identify regions that are co-segregating with affected family members. Most hereditary PCa family linkage studies have focused on European descent populations (59). Utilizing positional cloning with linkage analysis successfully linked hereditary prostate cancer gene 1 (*HPC1*) at 1q24-25, *HPCX* (Hereditary Prostate Cancer gene X) at Xq27-28, linkage at 8p22-23, *HPC20* (Hereditary Prostate Cancer gene 20) at 20q13, *HOXB13* (Homeobox B13) and others. Studies later identified linked hereditary PCa regions for the following candidate familial PCa susceptibility genes: hereditary prostate cancer gene 1 (*HPC1*) as *RNASEL (Ribonuclease L)*, *HPC2* (Hereditary Prostate Cancer gene 2) as *ELAC2 (ElaC Ribonuclease Z 2)* and 8p22-23 as *MSR1 (Macrophage Scavenger Receptor 1)* (87).

Since the advent of Virchows’ hypothesis, carcinogenesis has been observed as unresolved inflammation and innate immunity has been implicated in not only antiviral defense but also in prostate carcinogenesis (88). As a result of genetic ancestry, differences in innate immune response has led to investigation of essential genes, such as *OAS1 (OligoAdenylate Synthetase gene 1)/RNASEL*, that may play a role in response to pathogens and prostate cancer disparity (89, 90). Constitutively latent, endoribonuclease RNase L is activated upon binding to 2-5A molecules, produced by interferon induced *OAS* genes (91). If a cell is infected by a virus or affected by other stress factors (e.g. inflammation), activated RNase L will degrade self-RNA (rRNA, tRNA, mRNA) and non-self RNA (viral dsRNA). Degradation of RNA in the cell, by RNase L, results in stress mediated apoptosis, and this mechanism has been proposed to contribute to the tumor suppressor activity of this gene/pathway.

*RNASEL* is an endoribonuclease enzyme activated by innate immunity pathways *via* cellular stress induced by viruses, chemotherapeutics, oxidative stress, and inflammation. Following the establishment of *RNASEL* as *HPC1*, several studies began to report that *RNASEL* genetic variants may also be associated with sporadic PCa risk across the following population sample sets: Finnish (92), Swedish (93), Jewish (94), German (95), European American (87), and AA (96). The most common *RNASEL* variants studied are R462Q and D541E, which have shown significant association with PCa risk (96–98). Though many studies support *RNASEL* as a PCa predisposition gene, some studies report inconsistent association of *RNASEL* variants with PCa risk (59, 93, 99). It is important to note, small sample sizes of high-risk populations, especially Africans and men of African descent, contributed greatly to these inconsistencies.

Positive association between common *RNASEL* variants, such as rs486907 (R462Q), rs627928 (D541E) and intronic rs11807829, with increased PCa risk and or inflammation (100), prompted the need to investigate the potential impact of RNase L dysfunction in *in-vitro* models. Xiang and colleagues demonstrated the presence of *RNASEL* SNP, R462Q, led to decreased dimerization in the activation of RNase L, thus leading to decreased RNA degradation, up-regulation of inflammatory genes and evasion of apoptosis (101). Unfortunately, Caucasian PCa cell lines were used in these functional studies (e.g. PC3, Du145, and LnCAp) (101). Taken together, data suggest, rs627928 (D541E), rs486907 (R462Q) and rs1187829 (Intron) may impact function of RNase L by alteration of apoptotic and inflammatory gene regulation leading to evasion of apoptosis and increasing risk of PCa development in EA men. Conversely, the impact of common RNASEL variants and RNase L dysfunction, in the AA population, remains controversial. Therefore, investigating possible impact of common RNASEL SNPs, i.e rs486907 (R462Q), in high risk groups, such as AAs, may have important clinical significance.

*MSR1* binds to many chemically modified molecules ranging from bacteria to modified lipoproteins and *ELAC2* was predicted to have encoded for an evolutionary conserved, metal dependent hydrolase, hence leading to postulations of environmental effects on prostatic tissue and differentiation that occurs after exposure (87, 102). The exact mechanisms of *MSR1* and *ELAC2* have not fully been elucidated for the underlying associated causation with PCa. However, a meta-analysis of 8 studies evaluating common *MSR1* variants, stratified by race, concluded *MSR1* gene does not confer overall major PCa risk but may confer moderate risk in AA men (103). Later, Rennert and colleagues conducted an association study evaluating the role of 16 variants in *ELAC2, MSR1, RNASEL* with PCa risk using 1361 EA (888 cases and 473 controls) and 294 AA (131 cases and 163 controls) (87). Data revealed significant differences in the 3 hereditary PCa disposition genes’ allele frequencies by race. AA men homozygous for *MSR1* IVS7delTTA with negative family history of PCa were more likely to have low grade (OR (odds ratio), 2.9; 95% CI (confidence interval), 1.2-7.2) or late stage disease (OR, 5.2; 95% CI, 1.1-25.7). *RNASEL* Arg462Gln was associated with positive family history of high stage disease (OR, 14.8; 95% CI, 1.6-135.7) in AA men. Interestingly, *ELAC2* showed no significant association with PCa predisposition in AA men (87).

In recent studies, highly penetrant variants were identified in PCa genes critical for molecular and biological processes including *HOXB13* (Homeobox related transcription factor 13), *BRCA1 (Breast Cancer Gene 1)*, *BRCA2 (Breast Cancer gene 2)*, and DNA mismatch repair (MMR) genes*. HOXB13*, is a critical developmental gene that regulates prostate cell differentiation, cell growth, and functions as a tumor suppressor (104). Studies revealed novel *HOXB13* variant G84E was mostly found in families of Nordic descent from Finland and Sweden. Interestingly, the novel variant was observed in one AA case. Further analysis revealed both of his chromosomes were of EA ancestry at the site of the gene (105). Thus, more robust association studies are necessary to identify novel significant variants in AA men, who are a heterogeneous population.

*BRCA1* and *BRCA2* are tumor suppressor genes responsible for repairing damage to DNA and play an important role in cellular integrity. The *BRCA* genes are most notable for their essential roles in increased hereditary cancer risk of the breast and ovary. However, studies have also implicated these genes in PCa etiology (106, 107). Similar to the effect of *BRCA1/2* mutations in hereditary breast and ovarian cancer, *BRCA1/2* variants have been shown to be moderately elevated to increase PCa risk across populations (108). In the Ashkenazi Jewish population, the association of *BRCA1* variants (185delAG and 5382insC) and *BRCA2* variant (6174delT) and PCa risk were evaluated. The *BRCA2* variant was shown to confer a 3-fold increased risk of developing high grade PCa, while *BRCA1* variants conferred moderate elevated risk (109). In a separate study, Petrovics and colleagues sequenced DNA from 1240 PCa patients (Stage T2 (N=935); advanced PCa (50% T3-4 (N=189); and metastatic PCa (N=116)), of whom 30% of the cohort were AA. Results revealed AA patients more frequently carried *BRCA1/2* variants when compared to EA patients (4.6 *vs*. 1.6%, respectively). The study concluded that men harboring *BRCA2* pathogenic variants were more likely to progress to metastasis (110).

There is mounting evidence implicating germline mutated MMR genes with increased PCa risk (111). Mutations in MMR genes including *MSH2 (MutS Homolog 2), MLH1 (MutL homolog 1), PMS1 (PMS1 homolog 1), PMS2 (PMS homolog 2) or MSH6 (MutS Homolog 6)* subsequently give rise to a highly penetrant autosomal dominant predisposition cancer known as Lynch syndrome that increase risk for colon, endometrial, prostate, and other cancers. Several studies have shown deleterious mutations in these genes are causal of cancer development across populations (112). Matejcic and colleagues performed targeted gene sequencing, using a panel composed of 19 MMR precancer disposition genes, in 2,453 AA and 1,151 Ugandan Cases and Controls with prostate cancer. With OR’s (Odds ratio) ranging from 4 to 15 in the combined study sample of AA and Ugandan men, rare pathogenic variants within *ATM (ATM Serine/Threonine Kinase gene), BRCA2, PALB2 (Partner and Localizer of BRCA2) and NBN (Nibrin)* genes were observed to be associated with increased risk of aggressive disease (113). Interestingly, this study is the first to investigate contribution of rare pathogenic and non-pathogenic variants in MMR genes associated with aggressive disease in men of African descent. Positive association with rare pathogenic variants in men of African descent supports possible clinical importance of rare pathogenic variants in DNA MMR genes involved in PCa development. Taken together, further investigation of large indels, rare pathogenic and nonpathogenic germline variants in these or other DNA repair genes may ultimately identify high risk groups, who may benefit from increased screening, targeted prevention and or therapeutic approaches.

## Somatic Mutational Landscape

In addition to the strong evidence of heritable genes that increase PCa risk across populations, acquired mutations to germline DNA have also been implicated (114, 115). While 10-20% of cancer cases are hereditary, 80-90% are sporadic. Therefore, comprehensive analysis of somatic mutations is essential to elucidate key genes and gene variations that induce PCa development and exacerbate PCa incidence and mortality disparities. Given the strong association of certain somatic mutations with PCa progression and metastasis, many candidate genes, such as AR (Androgen Receptor) CAG/CGN repeats, *TMPRSS2*-*ERG* (Transmembrane Protease, Serine 2- ETS related gene) fusion, *LSAMP (Limbic System Associated Membrane Protein), APC (Adenomatous Polyposis Coli), ATM, BRCA2, KDM6A (Lysine Demethylase 6A), KMT2C (Lysine Methyltransferase 2C), KMT2D (Lysine Methyltransferase 2D), MED12 (Mediator Complex subunit 12), ZFHX3 (Zinc finger Homeobox 3), and ZMYM3 (Zinc Finger MYM-type Containing 3)* have been identified. Studies have shown somatic mutations, in these candidate genes, present with significant differences in frequency in men of WAA compared to men of European Ancestry.

Recent studies have investigated the consequences of CAG and CGN repeats, in the first exon of the *AR*, on androgen transcription activity as it relates to PCa risk (116). AA men often have shorter CAG and CGN repeats than EA men, and Zeegers and colleagues showed men with CGN repeats less than 16 have an increased PCa risk (117). However, in a cohort of Nigerian men, Akinloye and colleagues showed no correlation of CGN repeats and PCa risk (118).

Other studies have investigated the ERG oncogene fusion with the *TMPRSS2* gene which has been shown to be a pervasive variant in 40%-70% of PCa cases (119). Evidence suggest higher frequencies of *TMPRSS2-ERG* gene fusion in PCa tumors of EA men compared to AA men (120); although *ERG*-negative status in AA men shows association with higher-grade tumor indices and a less favorable clinical outcome (121). In addition, *TEMPRSS-ERG* acquisition appears to be inversely associated with aggressive PCa in tumors of Black South Africans (122). Taken together, data supports a link between *TMPRSS2‐ERG* status and PCa racial health disparity, beyond the borders of the United States.

Petrovics and colleagues conducted a comprehensive analysis of 435 genomic profiles of prostate tumors between AA and EA men. This study utilized whole genome sequencing (7 AA men and 7 EA men), FISH evaluations (101 men), and SNP array (320 men). Data confirmed previously recurring somatic variants detected in coding sequences of *SPOP (Speckle-type POZ protein), MED12, TP53 (Tumor Protein 53), KMT2C, ATM, CTNNB1 (Catenin Beta 1) and PIK3CB (Phosphatidylinositol-4,5-Bisphosphate 3-Kinase Catalytic subunit Beta)*. In addition, *PTEN (Phosphatase and Tensin Homolog)* and *ERG* alterations were significantly lower in AA men than EA men while frequency of inter-chromosomal rearrangements were higher in AA men compared to EA men. Interestingly, in addition to identifying the presence of recurrent deletion in CHD1 (Chromodomain Helicase DNA-binding protein 1), recurrent deletions in the LSAMP region (3q13.31) was more prevalent in AA men than EA men (123). *LSAMP* deletions have been associated with aggressive disease (124).

To further elucidate biological determinants that may play a role in PCa disparities, recent studies have investigated genomic alterations in prostate tumors derived from AA compared to EA men (125). Liu and colleagues performed targeted deep NGS (next generation sequencing) in 81 AA matched tumor-normal pairs, to analyze somatic mutations in 39 carcinogenic driver genes. In addition, performed a genome wide OncoScan for CNA (copy number alterations) in 171 AA tumors. Data showed >35% of AA men harbor damaging mutations in *APC, ATM, BRCA2, KDM6A, KMT2C, KMT2D, MED12, ZFHX3, and ZMYM3*, each with >1% of mutated copies. Also, more frequent deletions in specific regions of the genome such as: of 2p22.2, 4q34.3, 2q22.1–2, 15q15.1, 6q15, 8p21.2, 13q14.2, and 8q24.21 may lead to more aggressive prostate cancer characteristics in AA compared to EA. These frequent deletions include the genes *THADA (Thada Armadillo Repeat Containing)* (9.8% *vs*. 3.9%), and NEIL3 (Nei Like DNA Glycosylase 3) (9.8% *vs*. 3.7%), in Gleason 7 Tumors, *LRP1B* (LDL Receptor Related Protein 1B) (44.4% *vs*. 10.2%) and *BUB1B* (Bub1 Mitotic Checkpoint Serine/Threonine Kinase B) (33.3% *vs*. 4.1%) in Gleason 8 Tumors, and *MAP3K7* (Mitogen-Activated Protein Kinase Kinase Kinase 7) (73.1% *vs*. 40.6%), *BNIP3L (BCL2 Interacting Protein 3 Like)* (65.4% *vs*. 35.1%), and *RB1 (Retinoblastoma gene 1)* (73.1% *vs*. 50.9%) in Gleason 9 Tumors. Interestingly, AA harbored more frequent deletions, compared to EA, in *MYC (MYC Proto-Oncogene, BHLH Transcription)* with 22.8% *versus* 12.9% in Gleason 7 tumors, and 55.6% *versus* 18.4% in Gleason 8 tumors. Taken together, Liu and colleagues show deletion of *MAP3K7, BNIP3L, NEIL3 or RB1, or gain of MYC* significantly associates with both higher Gleason grade and advanced pathologic stage in AA men (125).

So far, many studies characterizing somatic mutational landscape in African descent populations have been conducted in AAs, but there is limited number of studies in African populations. Blackburn and colleagues assessed frequency of *TMPRSS2-ERG* fusions in Black patients with various ethnicity from South Africa and reported that *TMPRSS2-ERG* fusions were found much less frequently in Black patients (12.8%) than AAs or Europeans (122). Another study found a higher tumor mutational burden in tumors from South African Black patients than tumor from European patients (126). These studies provide support for tumor molecular differences in AA men compared to European men. Considering genomic and biologic diversity in Africa, a great tumor molecular variation may also exist, and there are critical needs for more tumor genomics studies in Africa to further examine tumor molecular variation.

## Tumor Microenvironment (TME) and Inflammation

The normal prostate gland microenvironment consists of epithelium (basal, stem, secretory luminal and neuroendocrine cells); stroma, consisting of the following: smooth muscle cells, fibroblasts, endothelial cells, and nerve cells; and the extra cellular matrix (ECM), a stromal component consisting of insoluble matrix and soluble factors (127). Studies have shown that insult to the prostate—either infectious or non-infectious—induces cellular stress and repeated genomic damage leading to immune upregulation and inflammation which prompts carcinogenesis (128). Both histological and clinical data confirm that chronic inflammation contributes to the onset and progression of cancer by altering the stromal phenotype leading to increased ECM remodeling and initiating epithelial mesenchymal transition (EMT) (129).

The latest reviews and meta-analyses demonstrate a strong correlation between a history of clinical chronic prostatitis and future PCa development in the general population (130). Conversely, Rybicki and colleagues purported clinical prostatitis was associated with a slightly decreased risk for prostate cancer in AA men; however, the number of samples are not sufficient to substantiate the claim (131). Therefore, there is an imperative need to increase the number of studies investigating immunity and inflammatory regulation in prostate tumor and adjacent TMEs for high risk under-represented populations. Kinseth and colleagues investigated differential gene expression associated with tumor tissue and adjacent ECM and observed 35% of significant pathways were associated with EMT as well as 25% associated with immune response pathways in AA men (132). Interestingly, most differentially expressed genes were associated with stromal tissue rather than tumor-specific tissue. ECM, Integrin family, and signaling mediators of EMT were all downregulated in AA compared to EA men. Gene expression differences in tissue reveal potential identification of PCa biomarkers such as PSPH (Phosphoserine Phosphotase) and CRYBBY2 (Crystallin Beta B2) and highlight the importance of tumor-adjacent ECM and stromal influence in prostate carcinogenesis for AA men (132).

While the identification of novel differential genes may elucidate essential mechanisms involved in chronic prostatitis leading to carcinogenesis, the elucidation of novel gene biomarkers for primary PCa diagnosis and prognosis is equally important. Panigrahi and colleagues performed exosome proteomics on serum extracted from AA men with PCa and identified 10 upregulated and 10 downregulated genes involved in inflammation and immune regulation. In addition, the study highlighted significant essential pathways for AA men with PCa. The top canonical pathways included Acute-phase response signaling (Rapid inflammatory regulated pathways), Compliment System (Immune regulated pathways), *LXR (Liver X Receptor)/RXR (Retinoid X Receptor)* activation (Numerous pathways including lipid, bile, and inflammation), *FXR (Farsenoid X Receptor)/RXR* activation (numerous pathways including lipid, bile, and inflammation), and Hematopoiesis from Pluripotent Stem Cells (133). Further investigation and validation of these results are needed since the E006AA-hT cell line was used and erroneously misclassified by race and disease type. Hooker and colleagues reported that E006AA-hT is neither a Black nor PCa cell line, but rather it is a renal cell carcinoma derived from a patient with 91% European descent (134). Taken together, amelioration of PCa treatment and diagnosis, is contingent upon proper investigation of high-risk underrepresented populations.

## Vitamin D Deficiency and Prostate Cancer Disparities

Vitamin D is a steroid hormone essential for normal physiological development, regulation of calcium homeostasis, and bone health (135). The synthesis of vitamin D involves a complex series of steps which begins with ultraviolet light (UV) initiating the conversion of precursor cholesterol molecule (7-dehydrocholesterol) into the vitamin D hormone precursor, cholecalciferol (vitamin D3). Subsequent conversion, *via* hydroxylation, takes place in the liver yielding 25(OH)D3. 25(OH)D3 hydroxylation in the kidney gives rise to the most active form of the vitamin D compounds, 1,25-dihydroxycholecalciferol or calcitriol. Vitamin D compounds are then transported in circulation after enzymatic binding to vitamin D binding protein (DBP) or multifunctional protein in the albumin family (Gc-MAF) (136). The synthesis of vitamin D initiates activation of Vitamin D Receptors (VDR), which form heterodimers with retinoid X receptors. Together, the heterodimer (*VDR-RXR*) complex recognizes vitamin D response elements (VDRE) in target genes and activation or represses numerous genes throughout the genome. This unique complex pathway synthesis of vitamin D synthesizes metabolites that are not only associated with calcium-related conditions, including osteoporosis (137), but also with non-calcium related conditions such as Covid-19 (138), breast, colon, and prostate cancers (139).

The anti-cancerous effects of vitamin D metabolites on tissue have been extensively studied. *In vivo* and *in vitro* functional studies have established vitamin D metabolites as exerting anti-proliferative, anti-inflammatory, pro-apoptotic, and pro-differentiating properties in a myriad of cell types (140). Meeker and colleagues showed that increased concentrations of vitamin D in *Smad3* knockout mice was beneficial in preventing inflammation-associated bacterially driven colon cancer and inflammatory bowel disease (IBD). Data suggest that the anti-inflammatory properties of vitamin D are due to the suppression of inflammatory markers, such as *NFkB (Nuclear Factor Kappa Beta 1) and MAPK (Mitogen Activated Protein Kinase)*, during the initiation of neoplasia in the colon (141). In breast cancer cells, Calcitriol initiates transcriptional repression of aromatase *via* promoter II in breast cancer cells and surrounding adipose tissue (142). In PCa cells, androgens elicit cellular growth *via* androgen receptor-mediated reactions. There is substantial evidence that establishes a crosstalk between calcitriol and androgen signaling of some PCa cells. Calcitriol seems to regulate the expression of androgen receptor which impacts cellular differentiation and growth inhibition (143). Epidemiological studies have shown that PCa patients with low serum vitamin D levels are likely to suffer higher PCa stage, grade, and mortality (144). Concurrently, vitamin D deficiency is common among AAs (145). This deficiency may largely be attributed to the presence of increased melanin inhibiting the skin from absorbing UV light from the sun, subsequently inhibiting conversion of free cholesterol into vitamin D3 in the body. While vitamin D deficiency is associated with increased PCa risk in men of African descent, functionally altering SNPs in the *VDR* may exacerbate the link to PCa risk and aggression. Jingwi and colleagues conducted an association study on 446 AA men from the AA Sporadic PCa study (AAPCA) and the vitamin D and PCa Risk in AA Men study including participants aged 35-93 years from the metropolitan area in Washington D.C. Data revealed *VDR* SNPs, rs731236 and rs7975232, to be significantly associated with PCa risk. *VDR* SNPs, rs731236, rs1544410, and rs3782905 were strongly associated with high PSA levels while *VDR* SNPs, rs1544410 and rs2239185, were associated with high Gleason score (146). Although the sample size was too small to confidently establish causal relationship between *VDR* SNPs, PCa risk and clinicopathological features (such as Gleason and PSA levels) in AA men, data suggests higher prevalence of select SNPs, in AA compared to EA men, may also contribute to the PCa disparity. Taken together, vitamin D metabolites have vast anti-carcinogenic properties. Given the innate vitamin D deficiency and concomitant burden of AAs suffering disproportionately from PCa risk and mortality, it is plausible to suggest increasing vitamin D levels in AAs, could mitigate the PCa disparity gap and elucidate novel pathways for therapeutic intervention.

## The Illusion of Inclusion in Prostate Cancer Clinical Trials

AA men diagnosed with PCa are more likely to suffer delayed treatment administration (4, 147) and less likely to receive definitive treatment (148). This delay contributes to worse outcomes. While co-morbidities such as obesity, diabetes, and hypertension occur more frequently in AA men and may alter the efficacy of optimal treatment (149), there is evidence to suggest that timely exposure to treatments can reverse this trend of poorer prognosis. In fact, recent studies report that AA men respond better than other men to several treatment options for advanced PCa. For example, the PROCEED study which treated over 1900 metastatic castration-resistant PCa (mCRPC) patients with sipuleucel-T reported longer median overall survival in AA *vs*. EA patients (150). In a separate meta-analysis comparing overall survival in 8,820 mCRPC patients treated with docetaxel plus prednisone, AA men fared better (151). This increased overall survival reported in AA men occurred despite younger age, worse performance status, higher testosterone, higher PSA, and lower hemoglobin described within this population. In a prospective, multicenter study of 100 mCRPC patients treated with abiraterone acetate and prednisone entitled ABI RACE, AA patients experiences greater median PSA progression-free survival rates as well as increased rates of PSA decline (152). While adverse events were similar in all patients, fatigue was notably lower in AA men.

While these reported results are certainly encouraging, there remains a tremendous opportunity to fully elucidate the mechanisms driving the racial differences in treatment outcomes for AA men with PCa. Without race-stratified clinical trials inclusive of an adequate amount of AA study participants, these improved therapeutic responses would remain elusive. AAs are less likely to be enrolled in clinical trials which limits opportunities to access cutting-edge treatment options (153). The barriers to clinical trial enrollment are many and include trust, access to healthcare, education, and communication gaps (39). Given the history of targeted medical victimization of AAs throughout U.S. history, it is easy to comprehend the widespread medical mistrust that continues to exist. Many examples of medical abuse in clinical research exist for this population, however the Tuskegee Syphilis Study may be the most prominent. From 1932-1972, the U.S. Public Health Service passively monitored the deliberate withholding of curative penicillin from approximately 400 AA men who had syphilis (154). The purpose of the modified experimental design was to observe the complete physiological demise incurred from the disease. The result was the unnecessary suffering of these men and the spread of syphilis in their families and communities. The impact of the Tuskegee experiment cannot be understated, and the psychological influence continues to trickle down to the decision-making process for many AA patients seeking medical care.

While the history of clinical trial enrollment and experience has been bleak for AAs in the U.S., there is hope for improvement. The recent spotlight on health disparities in COVID-19 outcomes and limited enrollment of study participants of African ancestry in associated vaccine clinical trials has brought this issue to the forefront (155). For researchers focused on tackling the overall problem of health disparities, this topic is not new. Therefore, there are several strategies in motion to actively address the need for increased participation by minority individuals. Tactics include increased funding to encourage health disparities research as well as financial incentives to cover the direct and indirect costs of trial participation for enrolled patients. In addition, concerned medical institutions are reducing travel concerns by expanding community sites to neighborhoods that are inclusive of individuals at high-risk of disease disparities (39).

The potential of improved survival outcomes in AA men with PCa is directly linked to increased efforts to design and implement race-stratified clinical trials. By including an adequate number of AA study participants, an immediate benefit will be provided to the high-risk patients receiving novel therapies. As an example, a current clinical trial combining talazoparib—an inhibitor of *PARP (Poly (ADP-Ribose) Polymerase 1)*—with androgen deprivation therapy and abiraterone acetate to treat patients with metastatic castration-sensitive PCa seeks to determine the effect of androgen receptor genetic variations on PSA nadir as a secondary outcome measure (156). Special attention and effort have been incorporated into the study protocol by the trial co-investigators (R.A.K. and L.W.B.) to ensure substantial accrual of AA men as well as men from other racial/ethnic groups to observe potential differential treatment responses associated with genetic variations. As inclusive trials such as these are completed, optimized plans of treatment based on observed and documented variations in treatment response will transition into standard clinical practice. Thorough assessments of modifiable and non-modifiable risk factors in race-stratified clinical trials will catapult the full potential for efficacious target treatments and advance precision medicine.

## Fulfilling the Promise of Precision Medicine

Since most GWAS-identified SNPs have not been replicated in AAs, there is a pressing need to identify these risk alleles in African descent populations. Elucidation of PCa genetics in African descent populations will provide improved insight on Polygenic risk scores (PRS), risk assessment and stratification, improved prognosis and ultimately improved outcomes in the population (157). However, heterogeneous associations due to differences in linkage patterns, population specific risk variants, differences in risk allele frequency, and many other factors affect transferability of the PRS, and the PRS developed based on GWAS in European populations is less accurate predicting disease risk (158, 159). Nonetheless, a multi-ancestry GWAS meta-analysis showed that African ancestry men have more than 2-fold higher mean PRS than European ancestry men illustrating overall increased genetic risk in men of African ancestry. Development and application of African ancestry specific PRS combined with PSA screening will help identify high-risk men who are likely to develop clinically significant PCa, while reducing overdiagnosis and overtreatment.

These current challenge in biomedical research is securing that the benefits of Precision Medicine are equitable across all populations. While recognizing that social/cultural, behavioral, and health care access factors are important causes of health disparities, many believe that genomic research can play important roles in reducing health disparities by understanding population differences in genetic risk factors, treatment response, and also interactions between gene and environment (social/cultural and lifestyle factors) and epigenomic variation. At the same time, some raised concerns about how genomics study findings may re-enforce the common misconception about race as a biological classification, as our understanding of genetic basis of health disparities increases. However, underrepresentation in research studies and the lack of genomic technology availability in racial/ethnic minority groups (e.g., lack of appropriate genetic testing for racial/ethnic minority groups) may widen health disparities.

## Author Contributions

JJ: Responsible for assisting with conceptual framework, drafting, editing and finalizing manuscript. LW-B: Responsible for drafting and editing. KB: Responsible for drafting and editing. SH: Responsible for Editing. RK: Responsible for conceptual framework, editing and drafting. All authors contributed to the article and approved the submitted version.

## Conflict of Interest

RK and LW-B have a current research grant funded by Pfizer.

The remaining authors declare that the research was conducted in the absence of any commercial or financial relationships that could be construed as a potential conflict of interest.

## Publisher’s Note

All claims expressed in this article are solely those of the authors and do not necessarily represent those of their affiliated organizations, or those of the publisher, the editors and the reviewers. Any product that may be evaluated in this article, or claim that may be made by its manufacturer, is not guaranteed or endorsed by the publisher.
